# Enhancing Intrapleural Hyperthermic Chemotherapy for Lung Cancer: Insights from 3D and PDX Models

**DOI:** 10.3390/cancers16203448

**Published:** 2024-10-11

**Authors:** Jung Young Shin, Mi Ran Lee, Kyung Ah Choi, Seok Whan Moon, Mi Hyoung Moon

**Affiliations:** 1 Laboratory of Medical Oncology, Cancer Research Institute, College of Medicine, The Catholic University of Korea, Seoul 06591, Republic of Korea; bearjy@gmail.com (J.Y.S.);; 2 DaNAgreen Co., Ltd., Seocho-gu, Seoul 06570, Republic of Korea; kyungah1985@gmail.com; 3 Department of Thoracic and Cardiovascular Surgery, Seoul St. Mary’s Hospital, College of Medicine, The Catholic University of Korea, Seoul 06591, Republic of Korea

**Keywords:** advanced lung cancer, pleural metastasis, intrapleural hyperthermic chemotherapy, PDX model

## Abstract

**Simple Summary:**

Intrapleural hyperthermic chemotherapy is a viable treatment option for malignant pleural effusion in advanced lung cancer. This study explored the effects of hyperthermic chemotherapy with cisplatin on lung cancer cells, including drug-resistant patient-derived tumor cells. By raising the temperature to 43 °C during treatment, we observed significantly enhanced antitumor efficacy and cancer cell death in both 2D and 3D cultures, as well as in a patient-derived xenograft model. The study showed that hyperthermia not only increased the efficacy of cisplatin but also promoted tumor necrosis and altered the expression of proteins associated with cancer cell survival. These findings suggest that hyperthermic chemotherapy may be a promising approach for improving treatment outcomes in patients with drug-resistant lung cancer.

**Abstract:**

**Background/Objectives**: Malignant pleural effusion (MPE) in lung cancer indicates systemically disseminated advanced lung cancer and is associated with poor survival. Intrapleural hyperthermic chemotherapy (IPHC) is a promising treatment for MPE; however, its biological basis is not fully understood. IPHC can enhance anticancer drug efficacy, particularly in drug-resistant cancers. This study investigated the effects of hyperthermia on cisplatin cytotoxicity in lung cancer cell lines, patient-derived tumor cells, and a patient-derived xenograft (PDX) model. **Methods**: Lung cancer cell lines (A549 and H2170) and patient-derived tumor cells were cultured in 2D/3D systems and treated with cisplatin under varying temperatures (37 °C, 43 °C, and 45 °C) and exposure times (5, 15, and 30 min). Antiproliferative effects were evaluated using LDH and CCK-8 assays. Optimal conditions identified in cell culture experiments were validated using a PDX model; tumor growth inhibition, delay, and protein expression were analyzed post-treatment. **Results**: Hyperthermia significantly enhanced the antitumor efficacy of cisplatin at 43 °C and 45 °C, with comparable effects under 15 and 30 min exposure. In the PDX model, IPHC showed increased tumor inhibition and necrosis and delayed tumor regrowth, particularly at higher cisplatin doses. Protein expression analysis revealed that hyperthermia decreased EGFR expression and increased levels of apoptosis-related proteins, including cleaved PARP and caspase-3. **Conclusions**: IPHC with cisplatin demonstrated enhanced antitumor efficacy in vitro models, particularly in drug-resistant lung cancer, indicating its potential as a valuable adjunct to existing treatment regimens for lung cancer and for improving patient outcomes in advanced lung cancer with MPE or pleural metastasis.

## 1. Introduction

Malignant pleural effusion (MPE) is the accumulation of exudates in the pleural space, accompanied by malignant cells or tissues [1]. Its pathogenesis is attributed to a disturbance in the Starling forces that govern fluid mechanics within the pleural space. This disruption is likely due to the occlusion of lymphatic stomata in the parietal pleura or the metastatic involvement of the lymph nodes, leading to impaired drainage and accumulation of pleural exudates. MPE is an important clinical concern as it is associated with poor prognosis, with a median survival of less than 7 months from the time of diagnosis. Symptoms such as chest pain, dyspnea, and cachexia substantially degrade the quality of life for patients [2]. MPE primarily results from metastatic diseases, with lung and breast cancers being the leading causes of MPE, accounting for approximately 50–60% of the cases [3]. In lung cancer, approximately 15% of the patients present with MPE at initial diagnosis, and up to 50% of patients develop MPE during the course of the disease [2].

Over the last few decades, significant progress has been made in the study of the pathophysiology, diagnosis, and imaging of MPE. However, the management of MPE is mainly palliative [4]. Currently, there are no efficacious treatment options for MPE; most treatment options are decided based on the symptoms, performance status of the patient, type of primary tumor, and response to systemic therapy. Close observation, repeated aspiration, indwelling catheter placement, and bedside or surgical pleurodesis are subsequently considered. Repeated pleural drainage and pleurodesis provide palliative care. Intrapleural hyperthermia or intrapleural hyperthermic chemotherapy (IPHC) has been used for the treatment of MPE in several centers, adapting from the principle of hyperthermic intraperitoneal chemotherapy (HIPEC) for malignant peritoneal carcinomatosis [5,6,7].

Giovanella et al. previously reported the tumoricidal effects of hyperthermia when tumors were exposed to 42.5–43 °C temperature, while the non-neoplastic cells were unaffected in cell culture models [8]. Hyperthermia also increases the cytotoxicity of many chemotherapeutic agents in cell culture and animal models [9,10]. HIPEC is widely used for controlling ascites and microscopic disease in peritoneal carcinomatosis resulting from gastrointestinal or ovarian cancer [11]. HIPEC has considerable survival benefits which have led to the application of the same treatment modalities for MPE and metastatic pleural disease [5].

Although there are limited data, IPHC has also shown promising results; however, the optimal temperature, drug concentration, and exposure time for IPHC are still unclear. In addition, both the treatment protocol and clinical results differ from center to center, with most studies being clinical exploratory studies based on a small number of patients, and biological evidence for IPHC is also poorly understood [12,13,14]. Cisplatin has been used to treat non-small cell lung cancer since the 1970s. In the era of immunotherapy and targeted therapy, platinum-based chemotherapy remains the major backbone of either perioperative or definitive systemic chemotherapy, especially in patients without drug-targetable drivers or perioperative systemic therapy [15,16,17]. Therefore, among the wide array of chemotherapeutic agents, cisplatin was selected for this study.

In this study, we designed an in vitro experiment to determine the optimal conditions for IPHC. We hypothesized that sizable tumor nodules in the pleural membrane hinder the therapeutic effects of IPHC. In addition, most preclinical studies have been conducted using 2D cells or cell lines, which do not accurately reflect the characteristics of the actual tumor microenvironment and pleural space [14,15]. Therefore, the objective of this preclinical study was to determine the optimal combination of temperature, exposure time, and drug concentration for the treatment of pleural metastasis and the associated MPE using patient-derived 3D cell models and a patient-derived xenograft (PDX) model.

## 2. Materials and Methods

**Figure 1:**
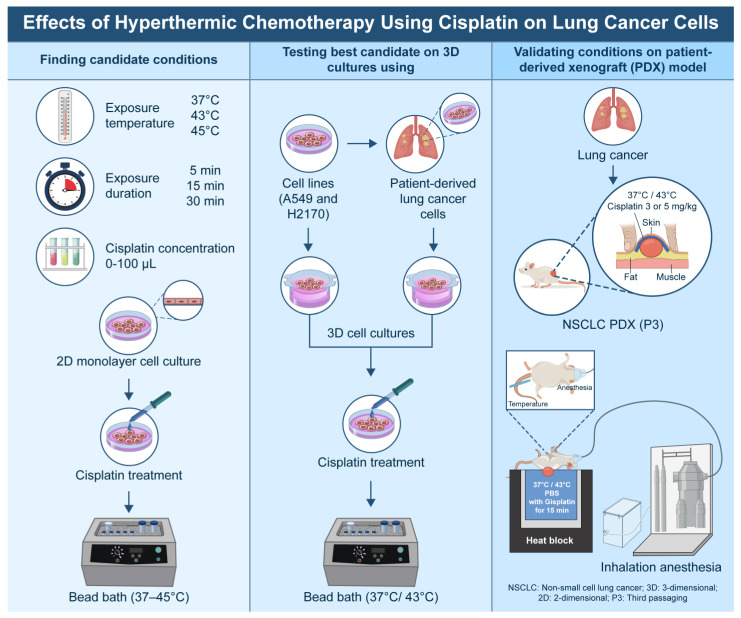
Flows of the experiments.

This study was conducted in accordance with the Declaration of Helsinki and was approved by the Institutional Review Board of the Catholic Medical Center, Republic of Korea (IRB approval no. KC18TESI0015; approval date: 8 March 2018). Informed consent was obtained from all patients. The overall flowchart of the experiment is shown in Figure 1.

### 2.1. Cell Lines, Patient-Derived Tumor Cells, and 2D/3D Cell Culture

The in vitro cell lines, including a hyperthermia-sensitive Chinese hamster ovary (CHO)-K1 cell line, as well as the human lung adenocarcinoma (A-549) and human lung squamous cell carcinoma (NCI-H2170) cell lines, were obtained from the Korea Cell Line Bank (Seoul, Republic of Korea). The cells were cultured in Dulbecco’s Modified Eagle Medium (DMEM, LM001-05; Welgene, Gyeongsan, Republic of Korea) supplemented with 1% penicillin–streptomycin (Gibco BRL, Invitrogen Co., Carlsbad, CA, USA) and 10 mmol/L 4-(2-hydroxyethyl)-1-piperazineethanesulfonic acid (HEPES) (Amresco, Solon, OH, USA). The cells were maintained in 100 mm plastic dishes (Eppen-dorf, Hamburg, Germany) and subcultured 2–3 times per week at 37 °C in a humidified atmosphere containing 5% CO2 and 95% air.

Patient-derived tumor cells were obtained with the patients’ consent during radical lung cancer surgery. The inclusion criteria were as follows: Patients were included if their tumor had a maximum diameter of ≥2 cm, had not received chemotherapy prior to surgery, and had not been previously diagnosed with another cancer. The collected samples were sectioned into 3–4 mm slices, and lung cells were isolated using 3 mL DMEM supplemented with 35 μL collagenase at 37 °C for 40 min. The following steps were then carried out to collect the lung cancer cells: Specimens were passed through a mesh filter (pore size: 0.2 μm) to remove any undigested tissue, the red blood cells (RBC) were lysed using an RBC lysis buffer (Roche, Cat. #27870800), and the cells were then washed with phosphate-buffered saline (PBS) and cultured in DMEM supplemented with 10% fetal bovine serum.

Cells extracted from the lung tissue were incubated with anti-TTF1-FITC (Abcam, Cat. #Ab76013, Cambridge, UK) at 1:50 dilution for 30 min, followed by washing with PBS. Flow cytometry analysis (FACS Calibur flow cytometer, Becton Dickinson Biosciences, San Jose, CA, USA) was performed to isolate lung cancer cells, which were then plated on Protinet Disk-Type plates (DaNAgreen, Seoul, Republic of Korea). A total of 5 × 10^5^ cells (equivalent to a cell pellet volume of 60 μL) were dispensed for triplicate cultures in a 3D cell culture system (Protinet™ scaffold, DaNAgreen, Seoul, Republic of Korea). Following the treatment, formalin-fixed, paraffin-embedded (FFPE) tissues were prepared and analyzed.

### 2.2. Preliminary Experiments: 2D and 3D

Considering the conditions in actual clinical practice and the findings of previous studies, the following conditions were selected for this study: drug exposure time of 5, 15, and 30 min; exposure temperatures varied from 37 °C (control) to 43 °C and 45 °C; and cisplatin (Sigma Aldrich, St. Louis, MO, USA) concentrations of 0, 10, 30, 50, and 100 µM in 2D cultures. The concentrations corresponding to IC_30_ and IC_50_ were selected for 3D cultures under hyperthermia conditions.

To ensure comparability, the combination treatment conditions of each temperature, exposure time, and drug concentration were applied to both the 2D and 3D cell line culture systems. The cells were plated in 96-well plates (Thermo Fisher Scientific, Waltham, MA, USA) at a density of approximately 3 × 10^3^ to 5 × 10^3^ cells/well and allowed to adhere for 24 h before treatment. Cells were treated with cisplatin (diluted in dimethyl sulfoxide) at each target concentration. The plates were treated in a hot basin at different temperatures and exposure times. Following treatment, the media was replenished, and the plates were cultured at 37 °C for an additional period of time before analysis. Based on these preliminary experiments, two optimal conditions each were identified for temperature, concentration, and duration of exposure. These cells were then replicated in a 3D cell culture system using patient-derived tumor cells.

### 2.3. Cell Proliferation Assay

The antiproliferative effects of each treatment condition were evaluated using the lactate dehydrogenase (LDH) and Cell Counting Kit-8 (CCK-8) assays. Cell growth inhibition was assessed using a CCK-8 assay (Dojindo, Kumamoto, Japan) following the manufacturer’s instructions. The absorbance was measured at 450 nm using an enzyme-linked immunosorbent assay (ELISA) reader. The drug concentration required to inhibit cell growth by 50% (IC_50_) was determined from the dose–response curve created using Sigma Plot software (version 8.0).

### 2.4. Validation with PDX Model

Within 24 h of lung surgery (for a total of 50 lung tissues), approximately 3 mm^3^ of cancerous tissue was transplanted subcutaneously into a four-week-old male NOD/SCID mouse (P0), following a pathologist’s visual inspection and confirmation of the tissue. If the volume of the implanted tumor reached 500 mm^3^, it was regarded as successful transplantation, and the tumor was excised (P1). Subsequent passaging of the tumor tissue (P2–P4) was performed.

The environmental conditions for all mice were maintained within the following parameters: 20–26 °C temperature and 50 ± 10% humidity in a 12 h light–dark cycle. The mice were provided with a gamma-sterilized diet (TD 2018S, Harlan Laboratories Inc., Indianapolis, IN, USA) and autoclaved reverse osmosis (R/O) water. Aspen bedding (PG-3; LAS Bedding, Soest, Germany) was also used.

All animal research procedures were conducted in accordance with the standards of the Laboratory Animals Welfare Act, Guide for the Care and Use of Laboratory Animals, and Guidelines and Policies for Rodent Experiments established by the Institutional Animal Care and Use Committee (IACUC) of the School of Medicine at The Catholic University of Korea (approval number: CUMS-2020-0152-02). In 2017, the IACUC and DOLA at the Songeui Campus of the Catholic University of Korea received an accreditation as a Korea Excellence Animal Laboratory Facility from the Korea Food and Drug Administration. The following year, the facility was awarded the AAALAC International full accreditation.

Once the tumor size reached 150–200 mm^3^, the mice were grouped, and growth curves were constructed for comparison. For the experimental treatment, the mice were anesthetized using a mild inhalational anesthetic agent (isoflurane) and then injected subcutaneously with a diluted solution of cisplatin (100 µL of 3 or 5 mg/kg at 37 °C and 43 °C, respectively). In clinical practice, intrapleural hyperthermic chemotherapy typically uses a heat exchanger and roller pump to perfuse chemotherapeutic agents into the pleural cavity, ensuring direct tumor exposure and consistent heat during treatment. To replicate this in our PDX model, we subcutaneously injected the drug into the tumor and immersed the tumor tissue in water maintained at 43 °C or 45 °C for a specified duration to simulate localized hyperthermia. The entire tumor was maintained at the target temperature for 15 min in a water bath. No alterations in the core (rectal) temperature of mice were observed throughout the course of treatment.

### 2.5. Measurement of Tumor Growth Inhibition and Tumor Necrosis

After the experiment, the tumor volume was calculated at two-day intervals using the formula (a × b^2^)/2, where “a” represents the length of the tumor and “b” denotes the width of the tumor. The percentage of tumor growth inhibition (TGI) was calculated using the following formula:

TGI (%) = 100 − [(mean tumor volume of the control group − tumor volume of the drug-treated group)/mean tumor volume of the control group × 100]

Tumor growth delay (TGD) was defined as the difference in the time required for the treated tumor to reach 1000 mm^3^ in size compared to the control. To determine the TGD, the tumor volume was measured on either day 15 or 25, contingent on the measurement schedule of the control group.

Tumor necrosis was quantified using hematoxylin and eosin (H&E) staining. The tumors were sliced into 2–3 sections of approximately 5 mm in length using a scalpel and then fixed in 10% formalin with rocking at 4 °C for five days. Following this, the sections were stained with H&E and fixed in 10% formalin for another 20 min prior to visualization. The presence of white and red areas would indicate the necrotic tissue and viable regions, respectively.

### 2.6. Protein Expressions

Protein expression was determined by immunohistochemical (IHC) staining and Western blot analysis. For IHC analysis, 4 μm thick paraffin-embedded tissue sections were deparaffinized with xylene. Endogenous peroxidase activity was inactivated by immersing the sections in methanol containing 3% hydrogen peroxide for 10 min, followed by washing with water and PBS. The sections were subjected to antigen retrieval by boiling in citrate buffer (Cat. #CBB500; ScyTek Laboratories, Logan, UT, USA). The tissue sections were incubated overnight with Ki-67 (Cat. #12202; CST) and cleaved caspase-3 (Cat. #9661; CST) antibodies at 4 °C, using a predetermined optimal dilution of 1:100. Subsequently, the slides were washed with PBS and incubated with a biotinylated secondary antibody (Polink2 plus HRP Rabbit DAB kit; Cat. #D41-125; GBI Labs, Bothell, WA, USA) for 10 min at room temperature. Subsequently, an additional washing step with water and PBS was performed, followed by the development of peroxidase activity using 3,3′-diaminobenzidine (DAB). The sections were then counterstained with hematoxylin and imaged at 200× and 400× magnification.

Western blot analysis was conducted on the proteins extracted from the tumor tissue in the PDX model and prepared in a sample loading buffer (0.125 M Tris-HCl [pH 6.8], 4% *w*/*v* SDS, 20% *v*/*v* glycerol, and 10% *v*/*v* 2-mercaptoethanol). Approximately 40 µg of protein was resolved by SDS-PAGE (10% gels) and transferred to PVDF membranes (0.45-µm pore; Cat. #IPVH00010; Merck Millipore, Burlington, MA, USA). The membranes were blocked for 1 h at room temperature with 5% *w*/*v* nonfat dried skim milk powder prepared in Tris-buffered saline (TBS) containing 0.1% Tween-20 (Cat. #IBS-BT005-1, iNtRON Biotechnology, Korea; Cat. #T1072, Biosesang, Republic of Korea). The membranes were probed overnight at 4 °C with antibodies against EGFR (Cat. #4267), TTF1 (Cat. #Ab76013; Abcam) at a 1:50 dilution, and apoptosis-related molecules caspase-3 (Cat. #9662; CST), PARP (Cat. #9532), and cleaved PARP (Cat. #5625) at a 1:1000 dilution. After washing, the membranes were incubated for 30 min at room temperature with the appropriate horseradish peroxidase-conjugated secondary antibodies. Subsequently, membranes were developed using the enhanced chemiluminescence (ECL^®^) Western blotting system (PXi4; Syngene, Cambridge, UK). β-tubulin (Cat. #2128; CST) was utilized as the internal loading control, and its presence was detected with specific antibodies (1:3000 dilution).

### 2.7. Statistical Analysis

All statistical analysis was performed using the SPSS software (version 20.0; SPSS Inc., Chicago, IL, USA). Data are presented as mean ± standard deviation. Statistical significance was determined using Student’s *t*-test for comparisons between means or one-way analysis of variance (ANOVA), followed by the Mann–Whitney U test. Statistical significance was set at *p* < 0.05. All experiments were conducted at least in duplicate. The main dataset is available as supplementary file (Dataset.xlsx). 

## 3. Results

### 3.1. Effect of Hyperthermic Chemotherapy in Cell Lines

**Figure 2:**
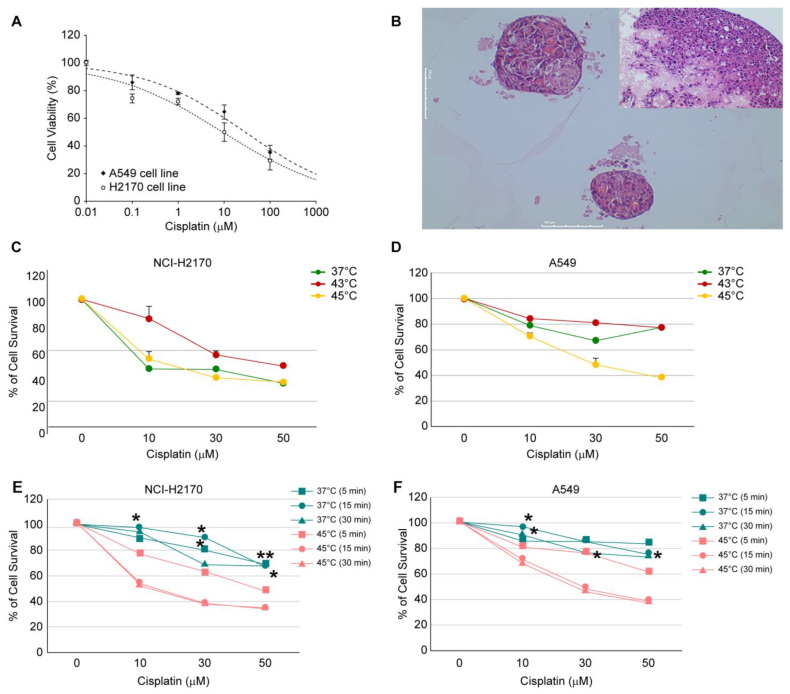
(**A**) IC_50_ values of A549 and H2170 cell lines treated with cisplatin. (**B**) 3D culture (Protinet-TP scaffold). A549 cells formed spheroids, reaching a diameter of 500 μm after 31 days of incubation (Hematoxylin and eosin [H&E] stain, 200× magnification). The inlet shows that H2170 cells formed spheroids after 49 days of culture (H&E stain, 200× magnification). (**C**,**D**) Viability of A549 (**C**) and NCI-H2170 (**D**) cell lines under varying concentrations and temperatures of cisplatin exposure (37 °C, 43 °C, 45 °C) for 15 min, with 5000 cells/well. Cisplatin treatment was conducted 7 days after seeding, followed by LDH assays. (**E**,**F**) Cytotoxic activity of cisplatin under various treatment conditions in H2170 (**E**) and A549 (**F**) cell lines. (* *p* < 0.05, ** *p* < 0.001).

To ensure comparability, each treatment condition was initially tested using 2D monolayer cultures and subsequently tested in 3D culture systems with lung cancer cell lines. Antiproliferative effects were evaluated 48 h after treatment using LDH assays. In the 2D cell culture systems, the IC_50_ values for cisplatin were 28.1 ± 0.1 µM in the A549 cell line and 9.2 ± 0.3 µM in the H2170 cell line, demonstrating a 3-fold difference in anticancer activity (Figure 2A). Both cell lines were then subjected to the 3D culture system for approximately seven days, with 5000 cells/disc (Figure 2B). Based on the IC_50_ results, cisplatin concentrations were set at 10, 30, and 50 µM.

No difference in anticancer effects were observed in cisplatin treatment at 5, 15, and 30 min at 37 °C in both the A549 and H2170 cell lines. When the exposure time was fixed at 15 min, the change in cell viability with concentration and temperature was as follows. In the A549 cell line, cell viability (%) remained similar across different cisplatin concentrations at 37 °C and 43 °C. However, the time-dependent anticancer effect was observed at 45 °C. At 45 °C, cell viability decreased by approximately two folds to 48.0 ± 6.2% and 38.3 ± 1.3% at 30 µM and 50 µM, respectively. In the H2170 cell line, hyperthermia reduced cell viability by approximately two folds at 43 °C and 45 °C compared to that at 37 °C (Figure 2C,D).

In the A549 cell line, treatment with 50 µM cisplatin for 15 min at 37 °C resulted in a cell viability of 72.8 ± 7.0%, which was not significantly different from the viability at 37 °C for 30 min (60.9 ± 1.8%). However, when the cells were exposed to cisplatin for 15 and 30 min at 45 °C, the anticancer effect was more pronounced than at 5 min at 45 °C. At 50 µM cisplatin treatment at 45 °C, cell viability was 38.4 ± 10.4% for 15 min and 37.1 ± 11.7% for 30 min (Figure 2C). In the H2170 cell line, cell viability was 47.6 ± 0.7% after 5 min of treatment with 50 µM cisplatin at 45 °C, which was lower than that observed at 37 °C for 30 min (68.4 ± 3.8%). At 45 °C, both 15 and 30 min of treatment resulted in comparable cell viability in both cell lines. These findings suggest that 15 min of hyperthermia is optimal for enhancing the efficacy of cisplatin in the A549 and H2170 cell lines (Figure 2E,F, Supplementary Figure S1).

### 3.2. IPHC in Patient-Derived Lung Cancer Cell Models

**Figure 3:**
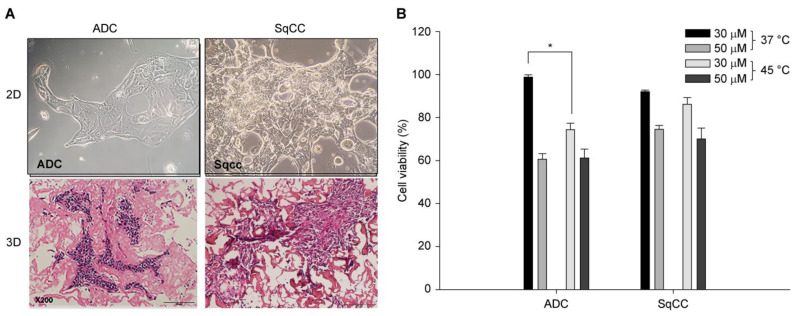
(**A**) Upper panels: 2D cultures of patient-derived lung cancer cells (OPTIMA CytoRek^®^, Optima Inc., Kawasaki, Kanagawa, Japan). Lower panels: 3D cultures of patient-derived lung cancer cells. In the H&E-stained sections of the 3D cultures (Protinet-disc, 200× magnification, light microscope), adenocarcinoma cells formed spheroid-like clusters, while squamous cell carcinoma cells formed sheet-like structures. (ADC: adenocarcinoma; SqCC: squamous cell carcinoma). (**B**) Cell viability assay for patient-derived lung cancer cells in a 3D culture system at different temperatures (37 °C vs. 45 °C) and cisplatin concentrations (30 μM vs. 50 μM), 15 min of exposure. * *p* = 0.05.

Tumor cells were isolated from the tissues of patients with lung cancer (adenocarcinoma and squamous cell carcinoma). The following characteristics were observed in the 3D cultures. First, more cancer cells were isolated from metastatic tissues than from primary lung specimens. Additionally, the growth rate of the inflammatory cells was higher than that of the cancer cells in the primary tissues. To estimate the growth rate of cancer cells in the 3D culture, the cells were identified using cell markers that were present in actual patients. In the case of lung cancer, 15.1% of the cell spheres extracted from the medium were identified as cancer cells in fluorescence-activated cell sorting (FACS) separation using the TTF-1 monoclonal antibody (1:50). Finally, the tumor cells were observed growing and forming spheroids in a 3D culture system (Protinet-disc) (Figure 3A).

In lung adenocarcinoma, cell viability with 30 µM cisplatin was 93.6 ± 7.2% at 37 °C and 71.3 ± 4.4% at 45 °C. These results confirm the presence of hyperthermic chemotherapy effect of 22.4% (*p* = 0.05, Figure 3B). However, at 50 µM, no significant difference in cell viability was observed between 37 °C (60.5 ± 2.6%) and 45 °C (61.0 ± 4.2%). In squamous cell lung cancer cells treated with 30 µM cisplatin, cell viability after 48 h was 87.5 ± 6.2% at 37 °C and 70.1 ± 6.1% at 45 °C, confirming a hyperthermic effect of 5.7% (*p* = 0.002). For cisplatin at 50 µM, cell viability was 83.1 ± 4.3% at 37 °C and 66.0 ± 4.9% at 45 °C (*p* = 0.017). After 72 h of treatment, no significant difference was observed in cell viability at the different concentrations (30 µM and 50 µM) or temperatures (37 °C and 45 °C). The effectiveness of hyperthermia was more pronounced in lung adenocarcinoma cells than in squamous cell lung cancer cells, although the difference was not statistically significant.

### 3.3. IPHC in a PDX Model of Patient-Derived Cells: A Case

The optimal experimental conditions determined in the 3D cell culture were validated using a PDX model created from patient-derived cells. The patient was a male diagnosed with lung adenocarcinoma (stage IIIA), who received first-generation EGFR-TKI (erlotinib) treatment for 60.1 months. Subsequently, tumor regrowth was observed on a follow-up chest computed tomography (CT) scan, prompting diagnostic surgery for mutation studies. Tumor tissue was obtained during this surgery, and the deletion and mutation of T790M was identified in the PDX tumor tissue, confirming drug resistance to erlotinib.

**Figure 4:**
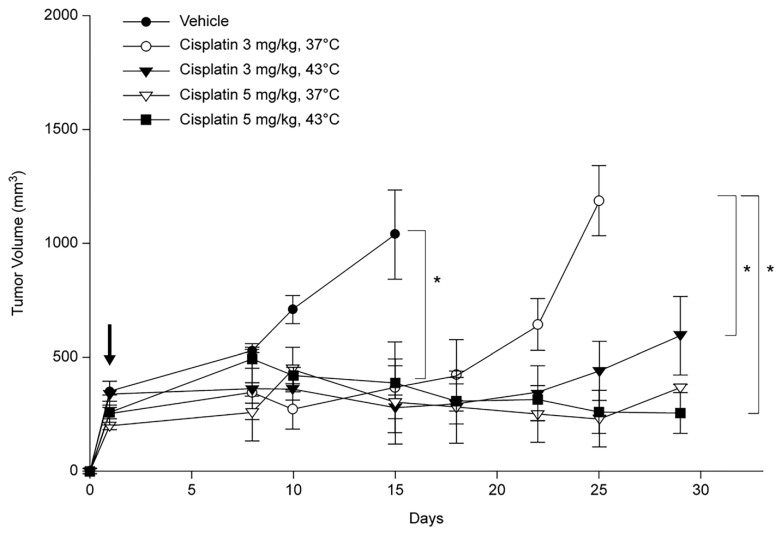
Changes in tumor volume after hyperthermic chemotherapy in a patient-derived xenograft (PDX) model of lung adenocarcinoma. Black arrow indicates the day on which hyperthermic chemotherapy was administered. * *p* < 0.05 (The comparison is based on day 15 and day 25).

The growth rate of the PDX model exhibited variability among the mice, and hyperthermic chemotherapy was administered when the tumor size reached a range of 202.73 ± 0.97 mm^3^ to 295.07 ± 29.51 mm^3^. Treatment efficacy was assessed over a 3-week period following treatment. Tumor size decreased at 37 °C and 43 °C with 3 mg/kg of cisplatin; however, tumor regrowth was observed after 17 and 21 days, respectively (Figure 4). Tumor size was either the same or decreased at both 37 °C and 43 °C, at doses of 5 mg/kg cisplatin.

We assessed the impact of hyperthermic chemotherapy on tumor growth inhibition (TGI) and tumor growth delay (TGD) based on a tumor size of 1000 mm^3^ (Table 1). At 37 °C, the TGD was 23.16 days with a cisplatin dose of 3 mg/kg but increased significantly to 107.32 days at 5 mg/kg under the same temperature conditions. When hyperthermia was introduced, the TGD became even more pronounced, increasing from 23.16 days at 37 °C with 3 mg/kg to 37.09 days at 43 °C with 5 mg/kg. At 5 mg/kg and 43 °C, the most significant TGI was observed on day 25, with a TGI of 78%, although the TGD slightly decreased to 95.30 days. No toxicities, skin-related side effects, or deaths were observed in any group at any dose.

### 3.4. IPHC-Induced Changes in Tumor Necrosis and Apoptosis-Related Protein Expression in PDX Model

**Figure 5:**
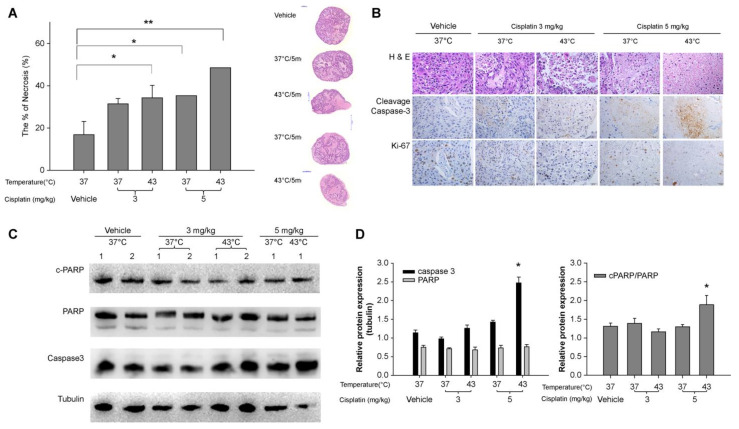
Expression of apoptosis-related proteins and extent of necrosis in the patient-derived xenograft (PDX) model constructed from tissues of a patient with lung adenocarcinoma were investigated. (**A**) Changes in tumor volume and light microscopic images of formalin-fixed, paraffin-embedded (FFPE) hematoxylin–eosin (H&E) staining (200× light microscopy, right panel). The area of necrosis (pinkish amorphous area) increased in comparison to the control group following the administration of hyperthermic chemotherapy. (**B**) Histological staining and immunohistochemical images (200×) of caspase-3 and Ki-67 in distinct experimental conditions are presented. (**C**,**D**) Western blot images (**C**) and a comparison of protein expression levels (**D**) in different experimental conditions. (*: *p* <0.05, **: *p* <0.001).

Tumor necrosis was confirmed by H&E staining of FFPE tissues, with analysis initiated three days after treatment. Highest efficacy was observed at 5 mg/kg of cisplatin with 15 min of exposure at 43 °C, with a 2.9-fold increase compared to that of the control group (48.5 ± 5.9% vs. 16.8 ± 6.4%, *p* = 0.02) (Figure 5A). Administration of 3 mg/kg cisplatin at 37 °C and 43 °C and 5 mg/kg at 37 °C resulted in tumor necrosis induction rates of 31.4 ± 2.5%, 34.3 ± 5.9%, and 35.3 ± 2.5%, respectively. These treatments demonstrated a 1.8- to 2.0-fold increase in necrosis compared to that of the control group, although the difference was not statistically significant.

Furthermore, IHC analysis was conducted on the tumor tissues from each group using antibodies against Ki-67, cleaved caspase-3, and other cell death-related proteins (caspase-3, PARP, and cleaved-PARP). The expression of these proteins was subsequently analyzed by densitometric analysis (Pannoramic MIDI; 3DHISTECH Ltd., Budapest, Hungary) and depicted in graphs. The frequency of Ki-67 protein expression, a marker of cell proliferation, was found to be comparable between the control and cisplatin 3 mg/kg groups, both in 37 °C and 43 °C. However, the frequency of Ki-67 protein expression in the cisplatin 5 mg/kg group was lower than that of the 3 mg/kg group. Furthermore, significantly lower expression was noted in the 43 °C hyperthermia group compared to the 37 °C control group (Figure 5B).

The expression of cell death-related proteins was altered at higher temperatures and drug doses. The cisplatin 3 mg/kg, 43 °C hyperthermia chemotherapy group exhibited a decrease in EGFR expression and a slight increase in cleaved PARP expression compared to that of the control group, displaying an expression pattern similar to that of the cisplatin 5 mg/kg, 37 °C hyperthermia chemotherapy group. In the group treated with cisplatin at 5 mg/kg and 43 °C, EGFR expression was similar to that of the control group and the cisplatin 3 mg/kg, 37 °C group. However, cleaved PARP expression was the highest, and caspase-3 expression was slightly increased (Figure 5C,D).

## 4. Discussion

The pleura is not just a membrane but is rather regarded as a functional organ. The pleura is covered by metabolically active mesothelial cells, which produce hyaluronic acid-rich glycoproteins, nitric oxide, and transforming growth factor β [18]. The thickness of the mesothelial cell lining is 4–5 um, and the cells are connected to each other by tight junctions on the luminal side and by desmosomes in the subpleural basal portion. The parietal pleura has abundant lymphatics, which are directly connected to the pleural space via the lymphatic stomata, and serve to absorb pleural fluid and substances, such as asbestos, that enter the pleural fluid. In contrast, the lymphatics of the visceral pleura are not directly connected to the pleural space and mainly serve as drainage sites for the pulmonary interstitial space [19].

The pleura is a common site of metastasis for most MPE arising from lung cancer, breast cancer, and lymphoma [18]. MPE is an indicator of poor prognosis and is classified as M1a stage in the 9th TNM staging system [20]. However, the extent and clinical presentation of pleural metastases or MPE can vary. In most cases, moderate-to-large amounts of MPE are accompanied by pleural metastasis, indicating an inoperable state. In such cases, the conservative management of pleural disease is recommended. Nevertheless, in some cases, a minimal volume of pleural metastases and an inconspicuous effusion may manifest clinically [21].

Treatments for MPE can be classified into three categories. The conservative treatment includes the drainage of pleural fluid, either through repeated thoracentesis or indwelling catheter placement. The second category includes more aggressive treatment of the pleural space with pleurodesis agents, such as talc, to prevent recurrent MPE. Finally, localized chemotherapy with or without targeted hyperthermia can be used to reduce pleural metastasis; this strategy has been attempted empirically but has not yet been standardized for use in MPE or pleural malignancy [4,22].

The role of hyperthermia in cancer treatment has demonstrated that treatment at temperatures between 40 and 44 °C is cytotoxic for cells in an environment with low pO2 and low pH. This is a common condition found within tumor tissues where blood perfusion is insufficient [23]. Hyperthermia induces protein denaturation and aggregation, which ultimately results in cell death via apoptosis or necrosis. Induction of hyperthermia (42–43 °C) to HeLa cells induces ER stress-mediated apoptosis and activates the calpain–calpastatin proteolytic system, which alters Ca2+ homeostasis that is also important in ER stress-related apoptosis [24]. In numerous in vitro studies, a temperature of 42–43 °C has been identified as lethal, whereas 40 °C is regarded as mild hyperthermia. Tumor cells are unable to increase blood flow in response to heat stress, which increases their vulnerability to heat damage compared to the surrounding normal tissues [25]. As most clinical practice, including intraperitoneal and intrapleural hyperthermic chemotherapy, is performed at temperatures up to 43 °C, with only the rare use of 45 °C for intrapleural hyperthermic experimental models. Therefore, we selected the test temperatures of both 43 °C and 45 °C in the preliminary stages of the experiment and tested at 43 °C in the validation study [6,13,14,22,26].

Local hyperthermia is a safe method of applying hyperthermia, whereas whole-body temperature has the potential to be dangerous [27]. Accordingly, experiments were designed to emulate actual localized hyperthermic chemotherapy in a PDX model and to replicate diverse pleural nodule sizes with inadequate blood flow in both the PDX and 3D cultures. This study demonstrated that 45 °C was an effective temperature for 3D cell culture and 43 °C hyperthermia was also effective in the PDX model. Furthermore, the anti-proliferative effect was more pronounced when combined with cisplatin, with the TGI% demonstrating a dose-dependent response and greater efficacy at higher temperatures.

Combining cytotoxic drugs and localized hyperthermia in cancer treatment is based on the experimental and clinical evidence that heat itself kills cancer cells by direct thermal toxicity and that heat increases the efficacy of chemotherapeutic drugs. Various drugs, such as doxorubicin [7], cisplatin [13], pemetrexed [28,29], or bevacizumab [30], have either been tested or used empirically. In the era of immunotherapy and other targeted therapies, cisplatin remains the major backbone chemotherapy for advanced lung cancers; therefore, cisplatin was chosen as the treatment agent in this study [16]. Recently, the results of an RCT using cisplatin as an IPHC were reported, and the dosage and exposure duration varied. The dose of cisplatin ranged from 40 mg/m^2^ to 200 mg/m^2^ [13,31,32]. In addition, the duration of exposure was quite long (1 h, 1–3 times/week) [12,13,22,31]. Considering the anatomy of the pleural membrane, IPHC may not be an entirely localized therapy. Some chemotherapeutic agents may be absorbed into the systemic circulation; thus, several toxicities have been reported, including leukopenia, thrombocytopenia, empyema, hemodynamic issues such as an increase in central venous pressure, cardiovascular collapse requiring vasopressors, and renal toxicities [31,33,34]. The reported morbidity rates are up to 30%, which signifies the importance of pre-clinical studies [35].

There are currently limited data on the pleura’s tolerance to hyperthermia. Animal studies on peritoneal membrane cells suggest that they can endure up to one hour at 43 °C [36]. However, in hyperthermia treatments in the pleura, the heart is involved, and authors have observed an elevation in the core temperatures and tachycardia when hyperthermia duration exceeds 20 min in clinical practice. To date, there is no standardization of hyperthermic intrapleural chemotherapy for pleural-based malignancies. However, the most recent update of recommendation about intrapleural hypertermic chemotherapy suggests the limit of temperatures as up to 40–43 °C [37]. Therefore, this study was designed with clinically applicable standards, using the time limits of 15 and 30 min to evaluate treatment effectiveness. Further research is needed to clarify the impact of treatment duration.

Cisplatin is a platinum-based compound that exerts its cytotoxic effects through a cell cycle-independent mechanism. It achieves this by covalently binding to the purine bases guanine and adenine in DNA. Upon entering cells via passive diffusion or specific transporters, cisplatin binds to DNA, inhibits DNA synthesis and mitosis, and induces apoptotic cell death. Additionally, cisplatin has been shown to generate reactive oxygen species, which can further damage cellular components such as lipids, proteins, and nucleic acids [38,39]. Since cisplatin does not undergo pre-metabolism in the liver, it can be inferred that the drug exposure in both the clinical IPHC and the PDX model used in this study was comparable. However, it is important to note that most bench research has been limited to malignant mesothelioma cells, with a paucity of basic research on other pleural malignancies, such as the various types of lung cancer. Therefore, further studies on the pharmacodynamics and pharmacokinetics of cisplatin are needed.

Our study revealed slight differences in the extent of the treatment response, with the H2170 cell line demonstrating a more pronounced response than the A549 cell line in 3D cultures (Figure 2). Furthermore, variability has been observed in patient-derived cell cultures and PDX models. This is probably related to innate thermotolerant and thermosensitive cell characteristics, as reported by Kalamida et al. [40]. In addition, mild hyperthermia about 40 °C can induce thermotolerance by alleviating heat-shock-induced ER stress response and apoptosis [24]. Another issue to consider for the best treatment option is the size of the nodule, which varies in clinical settings, and the required exposure time to ensure drug penetration must be determined on a case-by-case basis. The study by Li et al. revealed that the average treatment depth of the parietal pleural tumor increased by 1 mm for each 30 min of treatment, with an additional 1 °C increase in the inlet temperature. However, no further increases were observed when the treatment exceeded 120 min [26]. Although a direct measurement of the treatment depth was not performed in the present study, a reduction in tumor volume was observed as early as day 15. Moreover, a sustained reduction in tumor volume was observed until day 25, at 43 °C, when cisplatin 5 mg/kg was administered. This indirectly suggests that drug penetration may be effective even in large tumor volumes. This underscores the importance of the PDX model, which likely plays a pivotal role in patient-centered precision medicine.

The primary limitation of this study was its preclinical nature with a relatively small number of specimens. Nevertheless, we conducted multiple experiments on 2D, 3D, and PDX models, as well as on patient-derived cells. Next, while 43 °C was identified as the optimal temperature for 2D culture, we tested only 43 °C in the 3D and PDX models. This reflects the clinical situation and several clinical updates, the necessity of which will probably be further validated by several preclinical and clinical trials in the future [37].

## 5. Conclusions

This study provides compelling evidence supporting the enhanced efficacy of hyperthermic chemotherapy using cisplatin in both the 2D and 3D cell culture systems as well as in patient-derived xenograft (PDX) models. The results demonstrated that 43 °C, 15 min of exposure time, and 5 mg/kg of cisplatin in PDX model showed efficacy as HIPC. This effect was further validated in the PDX model, in which the hyperthermic conditions improved tumor growth inhibition, delayed tumor regrowth, and increased tumor necrosis, particularly at higher cisplatin doses. The observed alterations in the expression of proteins related to cell proliferation and apoptosis further support the mechanistic basis of the enhanced anticancer effects observed under hyperthermic conditions. Future studies should explore the detailed regimen, long-term outcomes, and potential clinical applications of hyperthermic chemotherapy in a wider range of cancers as well as its integration into existing treatment regimens for drug-resistant tumors.

## Figures and Tables

**Table 1:** Antineoplastic effect of hyperthermic chemotherapy.

Treatment	Dose (mg/kg)	Temp (°C)	Tumor Volume (mm^3^)			TGI% * (Day 25)	TGD ^†^ (1000 mm^3^)
			Day 1	Day 5	Day 25		
Vehicle	–	37	350.85 ± 42.42	1175.48 ± 196.02	1726.0 ± 507.69	–	14.48
Cisplatin	3	37	259.07 ± 29.51	368.71 ± 197.19	1079.39 ± 153.11	37.46	23.16
Cisplatin	3	43	337.79 ± 60.26	282.25 ± 52.98	673.97 ± 100.69	42.66	37.09
Cisplatin	5	37	202.73 ± 0.97	306.17 ± 188.60	232.95 ± 124.97	68.61	107.32
Cisplatin	5	43	257.76 ± 74.62	390.17 ± 73.96	262.33 ± 93.41	78.24	95.30

* TGI %. ^†^ TGD.

## Data Availability

The original contributions presented in the study are included in the article/Supplementary Material, further inquiries can be directed to the corresponding author.

## Supplementary Materials

The following supporting information can be downloaded at: https://www.mdpi.com/article/10.3390/cancers16203448/s1, Supplementary Figure S1. Bar graph showing viability of A549 (A) and H2170 (B) cell line under varying conditions and temperatures of cisplatin treatment. * and ** means *p* < 0.05. Dataset.xlsx contains the main experimental datasets.
