# Supplementary figures and images for: Enhancing Intrapleural Hyperthermic Chemotherapy for Lung Cancer: Insights from 3D and PDX Models
Source: Cancers (Basel). 2024 Oct 11;16(20):3448. doi: 10.3390/cancers16203448 (PMC11505734; doi:10.3390/cancers16203448)

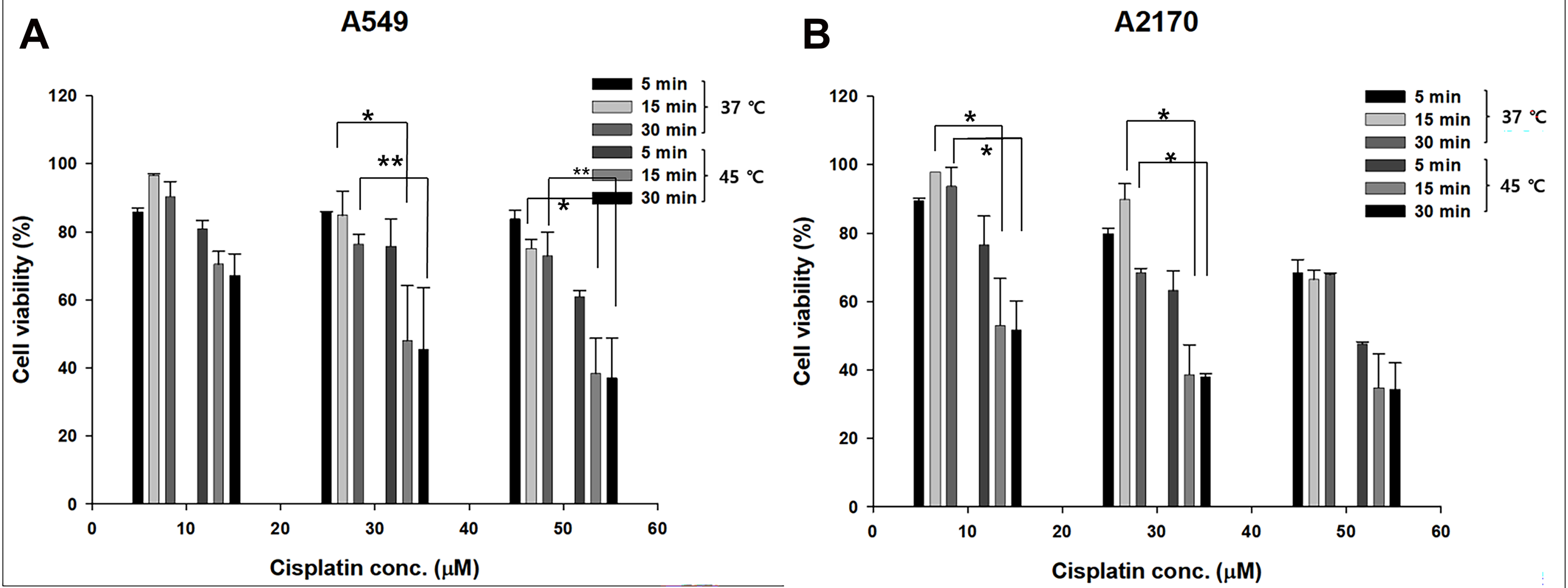

Supplement: Supplementary file 1 [file cancers-16-03448-s001.zip › cancers-3220533-supplementary.tif]
